# Georeferenced soil provenancing with digital signatures

**DOI:** 10.1038/s41598-018-21530-7

**Published:** 2018-02-16

**Authors:** M. Tighe, N. Forster, C. Guppy, D. Savage, P. Grave, I. M. Young

**Affiliations:** 1 School of Environmental and Rural Science, University of New England, Armidale, NSW 2351 Australia; 2 138 Toms Gully Road, Black Mountain, NSW 2365 Australia; 3 Archaeomaterials Science Hub, Archaeology & Palaeoanthropology, University of New England, Armidale, NSW 2351 Australia; 4 Sydney Institute of Agriculture, School of Life and Environmental Sciences, University of Sydney, Camperdown, NSW 2006 Australia

## Abstract

The provenance or origin of a soil sample is of interest in soil forensics, archaeology, and biosecurity. In all of these fields, highly specialized and often expensive analysis is usually combined with expert interpretation to estimate sample origin. In this proof of concept study we apply rapid and non-destructive spectral analysis to the question of direct soil provenancing. This approach is based on one of the underlying tenets of soil science – that soil pedogenesis is spatially unique, and thus digital spectral signatures of soil can be related directly, rather than via individual soil properties, to a georeferenced location. We examine three different multivariate regression techniques to predict GPS coordinates in two nested datasets. With a minimum of data processing, we show that in most instances Eastings and Northings can be predicted to within 20% of the range of each within the dataset using the spectral signatures produced via portable x-ray fluorescence. We also generate 50 and 95% confidence intervals of prediction and express these as a range of GPS coordinates. This approach has promise for future application in soil and environmental provenancing.

## Introduction

Determining the provenance or origin of a soil sample is of interest in soil forensics and many archaeological and environmental tracing studies^1,2^. The approach taken in these fields is usually a combination of high precision analysis and expert interpretation of the results^3^, which can be both highly technical and time consuming^4^.

Determining the provenance of a soil sample may never be absolute^5^, but an indication of origin can be useful in many instances, such as guiding police in the initial steps of an investigation^6^, determining archaeological sources of trade goods such as ceramics^1,3,7,8^, or potentially even informing biosecurity^9^.

Different statistical approaches are popular when analyzing soil or similar data for provenancing work. These range from Principal Components groupings of data points through to more novel approaches such as Lark and Rawlins^6^ development of a spatial likelihood function. These types of existing analyses are still mostly dependent on the quantification of particular physical or chemical soil properties in both reference dataset samples and the samples of interest. This is a time consuming and costly process. While rapidity of analysis is not essential when estimating provenance, it could be a distinct advantage in both time and financial savings, particularly when reference databases may require hundreds or even thousands of samples to ensure robust baseline data for comparisons.

Several rapid semi-quantitative analyses exist that can provide spectral signatures or elemental abundance data for soil samples. Equipment such as portable Visible to Near Infrared (Vis-NIR) and portable X-ray fluorescence (PXRF) spectrometers combine rapid and non-destructive analysis^10–12^. Visible to near infrared spectra in particular have been used to predict different soil properties such as soil carbon, pH, and mineralogy^10,13–15^. This is done using data mining techniques such as partial least squares regression, in which a large set of potential predictor variables are combined and compressed in multivariate space to produce a calibrated predictive regression^16^. To date, these combinations of spectral techniques and data mining approaches, have been used to predict soil provenance by first determining predicting soil chemical or physical properties, and then matching these properties to locations or soil types^17–20^. The success of this approach is due to the many spectral absorbance features in the visible to near infrared wavelength range that relate to different soil properties^10^. Similar multivariate approaches using PXRF estimates of elemental abundances have been applied in the discipline of archaeology and palaeontology^21,22^.

If determining the provenance of soil is the aim of the analysis, then it may be possible to make the approach more spatially explicit. It is a basic tenet of soil science that climate, parent material, and land use history combine at any given location to produce a soil with unique properties^23^. This uniqueness is reflected in elemental composition, mineralogy, and organic constituents of the soil^24^. Thus, it may be possible to combine rapid, non-destructive analysis that can detect one or more of these unique properties with data mining techniques to predict the actual provenance, or Global Positioning System (GPS) origin, of a soil sample of interest.

This study assessed the concept of predicting the actual GPS coordinates of samples by comparing a spectral signature with a reference set of spectra. Three data mining approaches were compared for each of two different rapid and portable analysis techniques – PXRF as a measure of elemental abundances in samples, and Vis-NIR as an amalgamated measure of minerals and compounds and other soil characteristics that respond in the measured wavelength range. The concept was assessed at two nested spatial scales within Australia.

## Results

### Individual Regressions

Table 1 displays the regression metrics for Eastings and Northings in both datasets using the vis-NIR and PXRF data. In one instance (prediction of Eastings using Vis-NIR) did the spline based method (EARTH) appear commensurate with the two other regression methods (PLS and PCR). Otherwise, the latter two methods appeared to be more accurate predictors of both Eastings and Northings. In the instances where EARTH was a poorer predictor than the other techniques, it overfits the training data (data not shown). Eastings were best predicted by both scanning approaches using the Local dataset (within 14–18% of the range of distance within the dataset). Northing predictions were generally more consistent and provided better predictions at the Farm level compared with Easting predictions. However the best fits were still found using PLS or PCR with either scanning (PXRF or Vis-NIR) method, to within 14–18% of the range of Northing distances within the dataset (Table 1).

**Table 1:** Model fit parameters and prediction results of Eastings and Northings using Vis-NIR and PXRF analysis of Australian Farm and Local dataset samples.

Scanning method	Dataset	Regression	Eastings				Northings			
			Components^1^	R^2^	RMSE (%)	AIC	Components^1^	R^2^	RMSE (%)	AIC
Vis-NIR	Australia – Farm	PLS	4	0.25	315 (20)	169	3	0.66	384 (15)	173
		PCR	10	0.07	350 (22)	184	4	0.68	376 (14)	174
		EARTH	8,6	0.24	318 (20)	—	6,4	0.09	632 (24)	—
	Australia – Local	PLS	10	0.72	426 (14)	244	14	0.48	1604 (23)	301
		PCR	18	0.72	427 (14)	260	15	0.54	1505 (22)	301
		EARTH	7,5	0.56	535 (18)	—	12,9	0.22	1973 (29)	—
PXRF	Australia – Farm	PLS	8	−0.13	387 (25)	183	4	0.67	370 (14)	174
		PCR	12	0.35	293 (19)	167	4	0.68	376 (14)	174
		EARTH	7,5	−0.69	473 (30)	—	13,9	0.51	461 (17)	—
	Australia – Local	PLS	12	0.62	496 (17)	254	17	0.75	1118 (16)	294
		PCR	17	0.73	420 (14)	257	17	0.71	1208 (18)	297
		EARTH	13,8	0.67	465 (16)	—	16,11	0.53	1530 (22)	—

^1^For PLS and PCR, components are the number of latent variables that minimized the adjusted general cross validation value on the training portion of data. For EARTH, the components are the number of terms and knot points automatically selected during the building of the piecewise splines of the model with 10-fold validation as per the methods section.

### Cumulative Probability Curves

**Figure 1:**
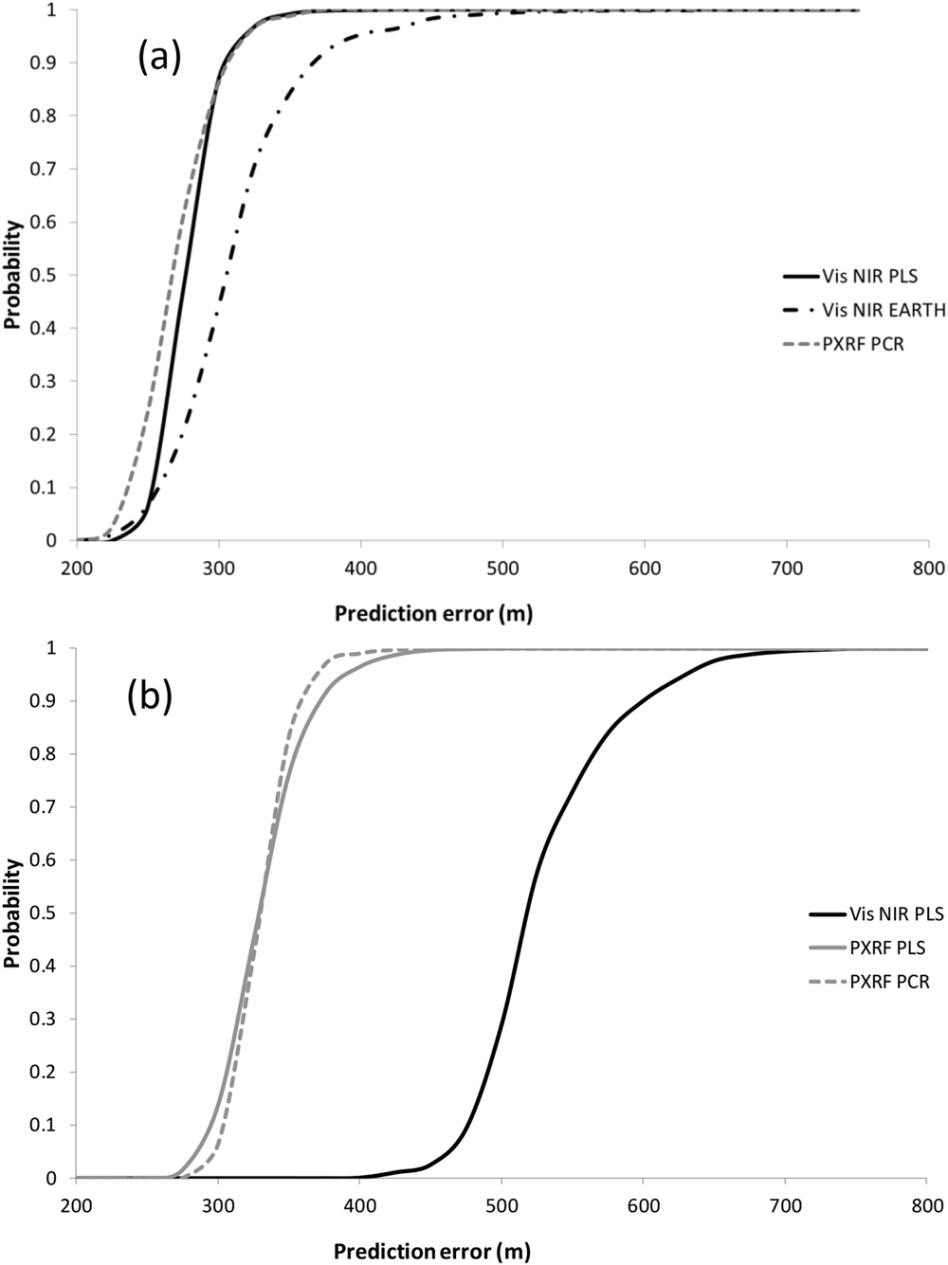
Cumulative probability distributions of distance predictions falling within a specified prediction error (m) for the Australian Farm dataset. The three regression approaches and instrument combinations as described in text are presented for (**a**) Eastings predictions and (**b**) Northings predictions. Vis-NIR = black lines, PXRF = grey lines. PLS = solid lines. PXRF = short dashed lines. EARTH = dash-dot lines.

Of the three potential predictors for Eastings and Northings at the Farm scale, PCR applied to PXRF data provided the best fit in terms of minimizing the prediction error at the cumulative probability levels of 0.5 and 0.95 (50 and 95%) (Fig. 1). Figure 1 also indicates that PLS of Northings applied to Vis-NIR data at the Farm scale included some instability – the RMSE from one regression prediction was much lower (384 m – Table 1) than indicated by the cumulative probability curve of 1000 simulations drawing from the Farm dataset.

**Figure 2:**
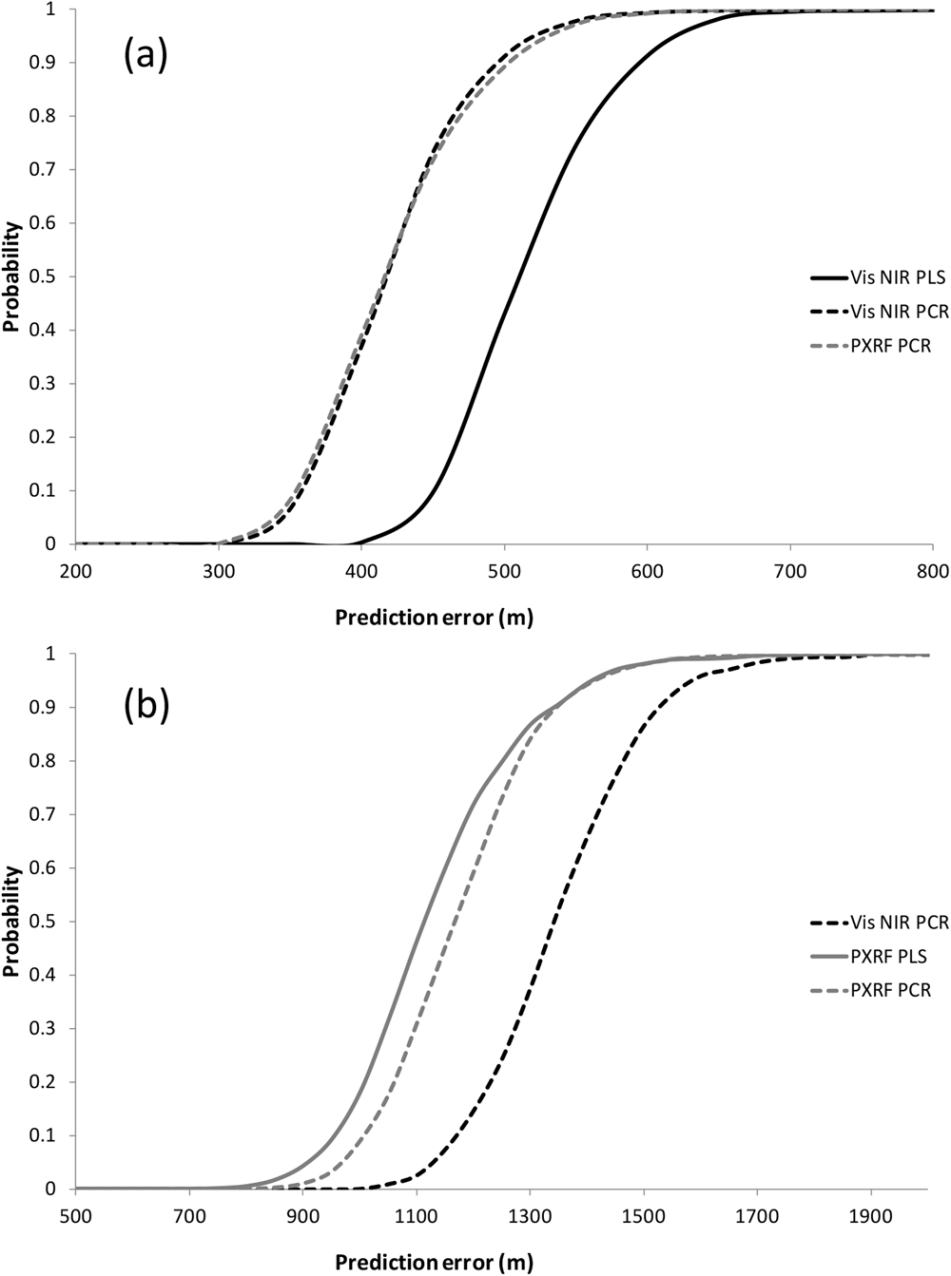
Cumulative probability distributions of distance predictions falling within a specified prediction error (m) for the Australian Local dataset. The three regression approaches and instrument combinations selected as described in text are presented for (**a**) Eastings predictions and (**b**) Northings predictions. Vis-NIR = black lines, PXRF = grey lines. PLS = solid lines. PXRF = short dashed lines. EARTH = dash-dot lines.

Figure 2 indicates that the ‘best’ predictor of Eastings at the Australian Local scale was also PCR applied to PXRF data. However, the Northings were best predicted at this scale by PLS applied to PXRF data.

### Dataset plots with predictive windows

**Figure 3:**
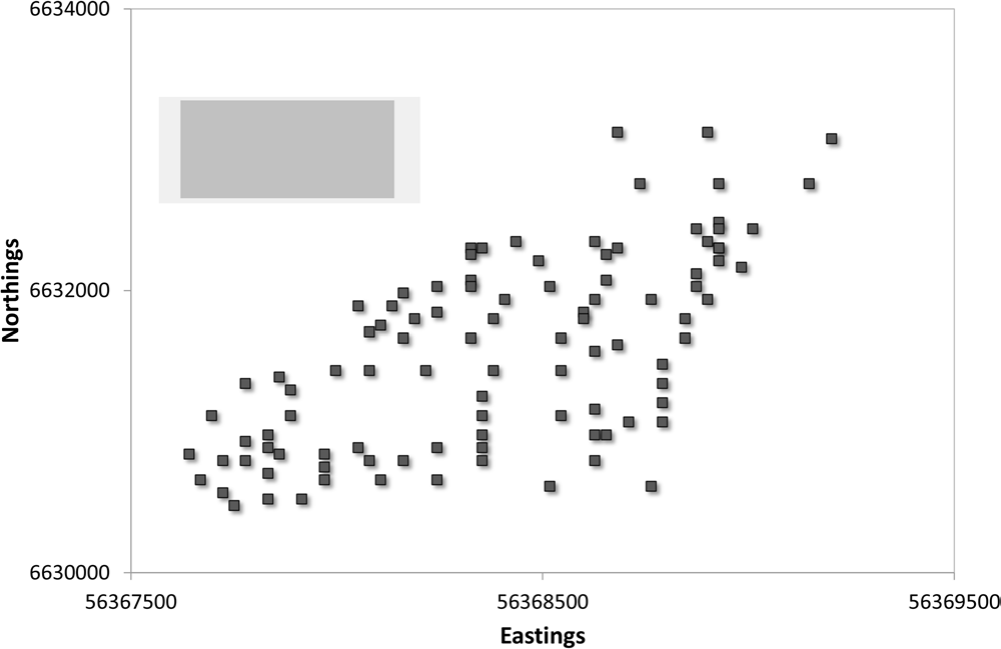
Sample points for the Australian Farm dataset, with the simulated average 50% (dark grey) and 95% (light grey) prediction space for the independent test samples overlain as the moving window of average prediction uncertainty as per text. As such the size of the shaded rectangles can be taken as graphical representations of model predictive performance. Prediction spaces were extracted from the probability distributions with the lowest cumulative error in Fig. 1.

**Figure 4:**
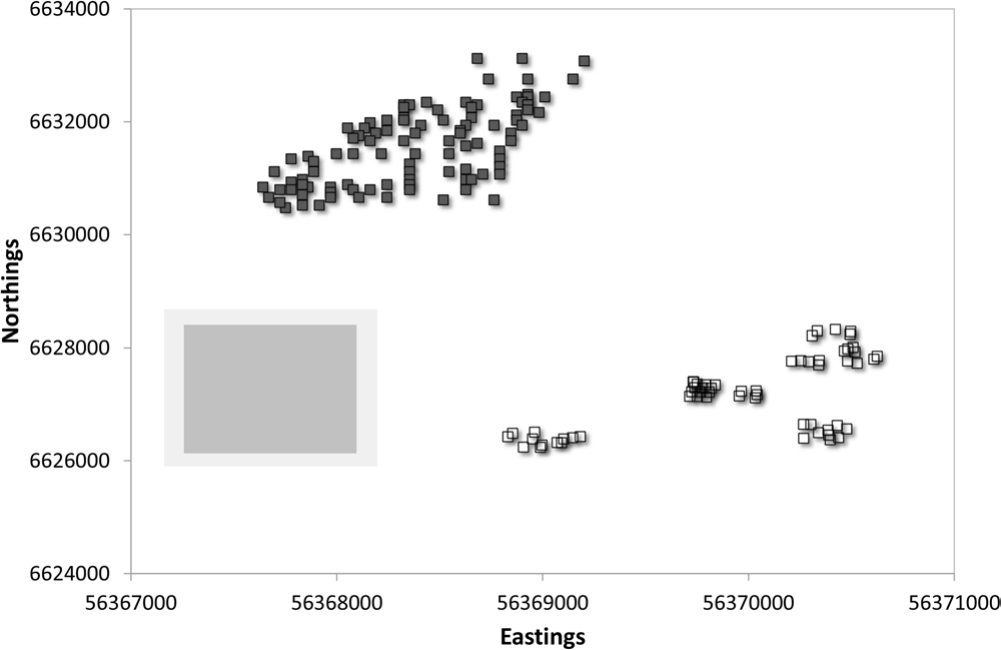
Sample points for the Australian Local dataset, with the simulated average 50% (dark grey) and 95% (light grey) prediction space for the independent test samples overlain as the moving window of average prediction uncertainty as per text. As such the size of the shaded rectangles can be taken as graphical representations of model predictive performance. Prediction spaces were extracted from the probability distributions with the lowest cumulative error in Fig. 2. The samples of the Local dataset that also comprise part of the Farm dataset are shown as filled dark grey squares.

Figures 3 and 4 display each dataset with the 50% and 95% confidence intervals of prediction. These rectangles can be considered ‘movable windows’ of average prediction confidence, as calculated from the test portion of the data. The relatively small difference in the 50% and 95% windows indicates the general steepness of the cumulative probability curves as depicted in Figs 1 and 2.

The predictive window in Fig. 3 was produced using the PCR regression of PXRF data for both Eastings and Northings. The lower precision of the Eastings predictions are represented by the longer x-axis of the predictive window compared with the Northings predictions. Figure 3 indicates that given an independent sample drawn from the area covered by the Farm dataset, the 95% confidence interval of prediction would be 640 m and 740 m on the Eastings and Northings axes, respectively. This equates to 41% and 25% of the east to west and north to south ranges respectively. The variable of most importance for both Eastings and Northings predictions was Fe, an element that is detected with high accuracy and precision by the PXRF^11^.

Figure 4 indicates the clustered yet overall spatially sparse nature of the Australian Local dataset, and the predictive window produced from the PCR regression of PXRF data for Eastings and the PLS regression of PXRF data for the Northings. Figure 4 indicates that given an independent test sample drawn from one of the areas covered by the existing dataset, then a 95% predictive confidence interval in the Eastings axis equates to approximately 950 m and 2000 m on the Northings axis, or 32% and 29% of the dataset range on the respective axes. This would provide delineation between the Farm samples and the additional Local samples, but with some potential overlap between the sample clusters in the bottom right hand corner of Fig. 4. As with the Farm dataset, the variable of most importance for both Eastings and Northings predictions at the Australian Local scale was Fe.

## Discussion

The objective of this study was to determine if it would be possible to combine rapid, non-destructive sample analysis with data mining techniques to directly predict the georeferenced location of an unknown sample of soil. This concept is based on the understanding that both soil pedogenesis and ongoing soil development are the product of climate, parent material, topography and other factors that are unique to any given location^24^. This is in essence the same concept applied in many soil provenancing approaches, but until now it has almost exclusively been applied to matching one or more soil characteristics or inferring spatial location indirectly^10,13,25^. In this study the concept was applied directly to georeferenced soil samples in two datasets to illustrate the feasibility of the approach.

Lark and Rawlins^6^ provided a rare exception to the usual indirect inferences of soil provenance in which they developed a spatial likelihood function for prediction of sample provenance based on disjunctive kriging and the multivariate structure of a geochemical dataset. This approach generated a probability of including the ‘true’ location given a sample signature. However, the authors indicated that at the time of the study the process was not easily automated and the necessary modelling was tedious. By considering two forms of rapid, non-destructive analysis and three different data mining/multivariate regression techniques that are already included in the freely available R software package^26^, we have shown that it is possible to generate Easting and Northing predictions of on average, to within 20% of the maximum distance within the considered datasets. When we considered the 95% prediction confidence intervals the error of these predictions did increase from less than 20% to 25–41% depending on the dataset and selected regression technique. While such accuracy is not in the realm required for true forensics applications, the ability to reduce an area of interest by ruling out areas with low probability has great potential in guiding more detailed provenance investigations across a range of disciplines. This appears particularly useful when local knowledge or soil expertise may be minimal.

Although we examined which variable was statistically ‘most important’ for each final predictive regression, the small size of the datasets precludes any in-depth analysis of which on-ground variables may be greatly affecting each model. However, it is noteworthy that both final dataset predictions of Eastings and Northings relied heavily on total Fe abundance in a relatively small area that is known to have a contrasting mix of granitic and basaltic parent materials which should generate, through pedogenesis, soils with contrasting total Fe signatures^24^. This suggests that given a reference dataset of appropriate sampling density and additional meta-data such as geological information, then estimates of variable importance may play a further role in improving provenance predictions. This also supports the use of Fe rich minerals in other provenancing studies in which different minerals can be diagnostic of climate related conditions, such as with the Chinese Loess Plateau^27^, and suggests benefits in combining this rapid approach with other, more specific provenancing techniques, including mineralogy, grain size and magnetic enhancement work^28–30^.

The generally superior predictive performance of PXRF data needs to be placed in the appropriate analysis context. Vis-NIR scans take 30 seconds (10 second scans in triplicate per sample) while the PXRF scans undertaken for this study were 18 minutes, not including the time taken to change the instrument parameters between analysis of ‘light’ and ‘heavy’ suites of elements. Reducing the PXRF analysis time to that of the Vis-NIR may see the latter analysis become the better provenance predictor. In addition, including some further data pre-processing of the Vis-NIR data (such as additional smoothing, examining the derivatives, or the log(1/reflectance) values) may improve the predictive capacity of this technique^10^. Similarly, longer PXRF scans may increase predictive power compared with results of this current study, as there is a well-documented relationship between X-ray scan time, accuracy and precision using PXRF^8,11^.

We have demonstrated how this concept of direct provenancing of georeferenced values can be applied due to the unique spatial nature of soil pedogenesis processes. This has already been proven numerous times in the related context of digitally determining soil chemical and physical characteristics that are helpful in inferring soil provenance^3,4,17–19^. There is much scope for development of this approach. In the vein of studies such as Viscarra Rossel and Behrens^15^, there are numerous data mining techniques that could be trialed and assessed. Similarly, there is a wealth of both rapid and more time consuming non-destructive soil analysis techniques, ranging from categorical morphological descriptors through to high resolution X-ray diffraction spectra^3^ that could be incorporated into such an approach. Many data mining techniques can accommodate both discrete and continuous data types, so different analysis types do not need to be examined in isolation – the use of complementary data may greatly improve spatial predictions, or permit verification of a provenancing conclusion from one method using another, independent method in an integrated approach as suggested by Nie and Peng^31^.

Another avenue of investigation is that of increasing rapidity of analysis versus any potential sacrifices in accuracy and/or precision of the predictions. In this study, we examined two rapid analysis techniques but undertook sample processing (drying and grinding) to increase analysis accuracy and precision at this proof of concept stage. Some rapid, non-destructive techniques are quite sensitive to additional factors such as water content and sample size (Vis-NIR in particular has spectral components that respond strongly to water^10^). Examining any loss of accuracy and precision of predictions as sample processing is reduced would help place this analysis approach in context – it may be a truly rapid, on-site application or it may be reserved for ‘rapid’ production of results compared to more time consuming, specialized analysis such as that reserved for true forensic evidentiary processes.

In any provenance study appropriate reference sample databases to compare unknown samples with is of paramount importance^10^. In this proof of concept study, we used two small, nested datasets as reference material. Any further investigation of the approach we have presented here would need to be undertaken in conjunction with the development or use of dataset(s) covering the initial area of interest at sufficient spatial resolution to make predictions sensible and useful. The successful application of the approach outlined here will depend upon the availability of a soil reference dataset matched to the appropriate scale of investigation (local, regional or even national), with appropriate caveats built into the interpretation of results. In particular, the influence of varying soil properties upon the robustness of the technique, such as organic matter or soil moisture ranges and influence on the regression approaches, need examination.

Similarly, how soil forming factors combine to produce a signature may potentially lead to ‘false positives’ if the approach is applied to areas without a robust reference set, or across areas that have similar digital signatures, needs to be examined. In such situations there exist a wide range of diagnostic techniques that are of use, from mineralogy through to isotopic provenancing approaches. In many instances, a combination of the approach we have demonstrated here along with other tools may be most beneficial, either using this rapid approach as a screening tool to reduce search areas, followed by more time consuming diagnostic approaches, or by using a diagnostic approach to eliminate areas across larger spatial scales (such as climatic zones or areas of disparate minerology) that pertain to local datasets, and then applying this rapid technique to the subset of data that remains. For example, Nie *et al*.^28^ used a combination of zircon U-Pb dating, heavy minerals and framework petrography to demonstrate where sediment from Northeast Tibet has been carried to via the Yellow River over very long time periods. Bird *et al*.^29^ applied a similar multi-faceted approach to long distance dust provenancing. Such approaches provenancing could be used in combination with the rapid screening approach here to further refine predictions of provenance^31^.

In addition, there is a large literature examining spatial correlation of predictions and ways to cope with such. While spatial correlation was minimal in this study, the checks of such as undertaken here are an integral part of such provenancing work. It is possible that modifications to typical regressive type approaches, such as the lag incorporations demonstrated by Lewis and Stevens^32^, or more contemporary regression-kriging analysis, possibly in combination with data mining approaches^33^, may be required in some instances.

These are not unique or new propositions, but important consideration in any soil provenancing approach that uses a reference dataset^6,14^. If the type of approach presented here ultimately proves useful in real-world contexts, then reference datasets may be developed for specific areas of interest, and statistical properties of such datasets, such as spatial correlations, should be determined. For example, border areas of neighbouring countries that experience biosecurity issues^34^ or criminal problems^35^.

In this study, we have developed a direct georeferenced soil provenancing approach that we consider useful in the context of numerous soil provenancing questions which all generically amount to the same query – i.e. how to exclude some areas or to narrow an area of interest in an examination. We have done this by applying one of the foundation concepts of soil science – that soil development factors combine uniquely at any given location to produce a spatially distinct soil – with rapid analysis that collects a signature related to numerous soil characteristics at once. This approach is amenable to different types of data and data mining approaches, and appears quite useful in reducing areas of interest in a given examination. It does remain to be determined if this type of approach could discern the likely provenance of ‘mixed’ samples, but we envisage such an approach as being potentially useful as a precursor to more rigorous and formal soil analysis for forensic purposes^7^ in certain instances. Like many provenancing approaches, an appropriate reference dataset for comparison to question samples will be of paramount importance in further applications.

## Methods

### Sample collection and dataset rationale

The collection of the two sets of data were designed to start with a relatively high density grid of samples at the farm scale and then add sparse additional samples at georeferenced points at what was considered a local scale (additional samples from nearby farms). These datasets are referred to as the Australian Farm and Local datasets respectively.

The Australian Farm dataset consisted of 100 samples taken on a grid across a 746 ha research station owned by the University of New England, Armidale NSW Australia (east to west range of 1561 m and north to south range of 2647 m). The Australian Local dataset consisted of the Farm dataset plus an additional 60 samples sourced from agricultural paddocks in the immediate vicinity of the University of New England, Armidale, NSW Australia (east to west range of 2984 m and north to south range of 6886 m). These additional 60 samples were collected on a random walk within paddocks (sampling stratified across any obvious slope differences). All samples for the Farm and Local datasets were taken from the 0–5 cm depth interval. Study location and sample points are displayed in Supplementary Figure 1.

All samples were georeferenced with a hand-held GPS device referencing the WGS84 datum and plotting to the UTM grid system (Eastings and Northings as the geographic Cartesian coordinates in this system, analogous with latitude and longitude in some other coordinate systems but expressed in metres). All samples were air dried and ground by hand to <2 mm prior to analysis.

### Vis-NIR

Approximately 2 g of each of the ground samples was placed in a small plastic petri dish for analysis. The diffuse reflectance spectra was measured using the Terraspec vis-NIR spectrometer with a spectral measurement range of 350–2500 nm (ASD, Boulder, Colorado USA). A thin plastic film was used to cover the contact probe to allow close contact between the sample and probe (rather than scanning from below through a transparent sample container^15^). To compensate for the added interference of the plastic film, the spectrometer (with plastic film in place) was calibrated against a white Spectralon calibration panel before each sample reading. Scanning occurred at sampling intervals of 1.4 nm for the spectral region 350–1000 nm and 2 nm for the region 1000–2500 nm. The maximum spectral resolution was 3 nm FWHM at 700 nm. Each sample was analysed three times (consisting of 100 internal replicates per scan) and an average spectra taken. Sample scanning time was 10 seconds. Spectra were collected in the associated equipment software R3 and were exported in the associated software TSG7 as csv files for statistical analysis. Data points between spectral resolutions were interpolated automatically during data processing, resulting in 2150 variables (wavelength readings) per sample. Each candidate variable was expressed in arbitrary reflectance units scaled between 0 and 1.

### PXRF

Ground samples were prepared as described previously^11^. Briefly, samples were placed in a small polyethylene container and sealed with a sheet of thin polyethylene film. Samples were analysed with a Bruker Tracer SD PXRF spectrometer. The instrument used a Rh miniature X-ray tube equipped with a Si-Pin detector with an active detection area of 7 mm^2^ and a resolution of 170 eV (determined at the Mn Kα edge). Samples were scanned in triplicate under two different PXRF settings. Low-Z elements (elements Na to Fe) were determined under vacuum conditions via a scan at 15 keV and 20 µA, and medium to high-Z elements (elements Fe to Pb) were determined via a scan at 40 keV and 30 µA using a 0.152 mm Cu, 0.025 mm Ti, 0.305 Al filter in the incident x-ray path. Scans were conducted at a 180 second live count per setting resulting in a total scan time of approximately 18 minutes per sample (including triplicates). All results were collected as raw spectra in the equipment software (S1PXRF 3.8.30) and converted in Spectra 7.2.1.1 to counts (relative abundance estimates of individual elements) using the net area under each elemental response curve after Bayes deconvolution. Abundance values were exported as csv files for statistical analysis, resulting in 21 variables (elements) per sample. Thus, the candidate variables were the elements Al, Br, Ca, Cr, Cu, Fe, K, Mg, Mn, Na, Nb, Ni, P, Rb, Si, Sr, Ti, V, Y, Zn, and Zr expressed as relative abundance estimates. Elements were selected based on standard recommendations for screening soil samples with PXRF. No attempt was made to reduce the elemental dataset by comparison to detection limits or potential interferences between elemental peaks. Rather, all calculated abundances were included as spectral responses of low elemental certainty are still potentially unique signals pertaining to a sample’s origin.

### Statistical analysis

Both Vis-NIR and PXRF data were analysed using three different data mining techniques aimed at reducing high dimensionality data to a useful predictive regression; partial least squares regression (PLS), principal components regression (PCR), and enhanced adaptive regression through hinges (EARTH).

Partial least squares regression is a popular chemometric technique for constructing predictive regressions from datasets in which the number of predictive variables may outnumber the samples, and/or when predictive variables are highly collinear^16,36^. Partial least squares regression compresses the data and can be considered a shrinkage method – selecting orthogonal factors (latent variables) from the higher dimension set of possible predictors by maximizing the covariance explained between predictors and response. Principal components regression is a very similar technique but does not include information from the response variable when selecting predictors (focusing on the variance of predictors, rather than covariance between predictors and response). While PLS is considered to have an advantage over PCR, in practical applications the differences are often slight and there are some situations in which PLS may increase the variance of individual regression coefficients^36^.

Enhanced adaptive regression through hinges is an application of the multivariate adaptive regression splines approach. It is a non-parametric multivariate regression approach in which the regression is built upon a series of splines. The parameters of the individual splines are determined via the data properties and ‘splits’ in the data structure that maximize the variation explained. The individual models are then combined into one additive regression. For further detail see Friedman^37^.

The dependent variables in each instance were the Eastings and Northings of the samples within each dataset. Each data mining technique was undertaken using each dataset and each of the dependent variables in turn. For each analysis, 30% of the dataset was randomly assigned to a test group to assess the predictive regression. Partial least squares and PCR were undertaken using the pls package^36^ in the statistical programming software R 3.2.2^26^. For both regression techniques, the appropriate number of latent variables was determined by examining the Adjusted General Cross Validation (GCV) estimates across models with a range of latent variables (1–35) and selecting that which minimized the GCV of the training portion of the dataset. The EARTH analysis was undertaken using the earth package in R^38^. For the EARTH analysis a 10-fold cross validation was used to fit the model to the training portion of each dataset with automated optimized selection of the number of terms and spline knots for the piecewise functions used to build the additive predictive regression. The final number of latent variables and the number of terms and spline knots for the EARTH analysis can be found in Table 1.

Following the above modelling, the predictive capacity of each selected model was then assessed on the test portion of each dataset using both the generalized R^2^ and the root mean square error of prediction (RMSE) values^39^. Root mean square error values were also expressed as a percentage of the total range of variation in the dataset (i.e. expressed in metres, and in percentage of total range of metres in the dataset). Akaike’s Information Criterion (AIC) was also computed for PLS and PCR analyses to provide information on parsimony. A directly comparable form of AIC does not exist for EARTH and thus was not calculated.

As each of the regression models may produce different results and possibly different degrees of spatial correlation depending on the subset of data randomly selected for cross-validation, the stability of the above predictions for each dataset were further assessed and cumulative probability distributions of prediction error were generated via simulation. Within each dataset analysis, the three regression models with the combination of the lowest AIC (for PLS and PCR), highest R^2^ and lowest RMSE values for each dependent variable (Easting and Northing) were recalculated after randomly drawing 70% of data from the training portion of each dataset. The generated predictive regression was then applied to the test set of data and the errors in prediction calculated. This process was repeated 1000 times and cumulative probability distributions of prediction error generated. These probability distributions were plotted and assessed graphically to further determine the best regression model to use for predicting Eastings and Northings within each dataset (Figs 1 and 2).

The ‘best’ predictors of Eastings and Northings were selected based on these curves (i.e. those curves with the steepest slopes and overall lowest prediction error across the probability space of 0–1). The 50% and 95% confidence intervals of prediction were estimated from these curves and plotted graphically in association with the respective dataset. Thus, Figs 3 and 4 show the sample points of the two datasets (including the spatial context of the Farm samples in relation to the additional Local samples) as well as two ‘average’ windows of sample prediction. These windows or rectangles represent the spatial range of Eastings and Northings that correspond to the average 50% and 95% prediction interval of all GPS values drawn from the test datasets. These can be interpreted as the average estimate of prediction confidence intervals when all test samples are considered via the 1000 iterations of prediction and thus graphically represent both the average prediction interval for the approach, and a visual representation of model performance. Practically these can be interpreted as ‘movable windows’ of prediction intervals that can be mentally placed over any part of the plotted dataset in lieu of individual sample estimates of prediction (which will provide estimates of prediction windows for individual samples rather than an indication of overall performance of the model when applied to the test set of data).

### Variable importance and additional analysis

The three data mining techniques were interrogated to develop an indication of the statistical importance of individual variables to the overall multivariate regression. For the final, ‘best’ predictors of Eastings and Northings for each dataset, variable importance was determined using the loadings function in the pls package for PLS and PCR^36^, and the drop1 function in the earth package for EARTH^38^. Only the most influential variable for each model is reported, so as to demonstrate the possibility of this type of model interrogation but not to place a large amount of emphasis on individual variables in this proof of concept study.

As multivariate calibrations using Vis-NIR data are often improved using the derivative of the spectra rather than the spectra itself, Vis-NIR data from the Local dataset were analysed both as raw absorbance spectra and as the first derivative of the spectra. The first derivative was calculated using the Savitzky-Golay algorithm with a filtering window of 10 data points and a quadratic smoothing term^40^. No improvements in the calibration results were found using the first derivative of the Vis-NIR data and these results are not discussed further.

In addition, the ‘best’ predictors as used to generate Figs 3 and 4 were also investigated for spatial correlation. As the ultimate goal of the analysis was to predict spatial locations as Eastings and Northings, the typical definition of spatial correlation in this instance was analogous to the more general interpretation of correlation between predicted results (heteroscedasity of predicted values). In addition, the concept of spatially correlated raw spectra cannot be defined without first applying a shrinkage method such as PLS (as each original data point is actually comprised of thousands of points). Thus, two approaches were taken to assess ‘spatial’ correlation. Firstly, predicted values were examined for heteroscedasticity via standard diagnostic plots. Secondly, using an approach similar to Overmars *et al*.^41^ and Lewis and Stevens^32^ we generated the variograms (omnidirectional) for the errors in the Eastings and Northings predictions using both the Farm and Local datasets using the variog function in the geoR package^42^. The Eastings predictions at the farm level showed some weak spatial correlation for samples <400 m apart which was considered minimal in this instance (Supplementary Figure 2). All other predictions showed no discernable spatial correlation.

### Data availability

The datasets generated and analysed during the current study are available from the corresponding author on reasonable request.

## Electronic supplementary material

Supplementary Material

## References

[1] Grave, P. *et al*. Ceramics, Trade, Provenience and Geology: Cyprus in the Late Bronze Age. *Antiquity* **88**, 1180–1200 (2014).

[2] Smith HG, Evrard O, Blake WH, Owens PN (2015). Preface - addressing challenges to advance sediment fingerprinting research. Journals of Soils and Sediments.

[3] Fitzpatrick, R. W. & Raven, M. D. Guidelines for Conducting Criminal and Environmental Soil Forensics Investigations: Version 7.0. 39 (Centre for Australian ForensicSoil Science, Adelaide, 2012).

[4] Robertson, J. Chapter 1. ‘Soils ain’t soils’: Context and issues facing soil scientists in a forensic world in *Criminal and Environmental* Soil *Forensics* (eds Ritz, K. Dawson, L. & Miller, D.) 3–12 (Springer Science + Business Media B.V., 2009).

[5] Aitken, C. G. G. Chapter 3. Some thoughts on the role of probabilistic reasoning in the evaluation of evidence in *Criminal and Environmental* Soil *Forensics* (eds Ritz, K. Dawson, L. & Miller, D.) 33–47 (Springer Science + Business Media B.V., 2009).

[6] Lark RM, Rawlins BG (2008). Can we predict the provenance of a soil sample for forensic purposes by reference to a spatial database?. European Journal of Soil Science.

[7] Fitzpatrick, R. W., Raven, M. D. & Forrester, S. T. Chapter 8. A systematic approach to soil forensics: Criminal case studies involving transference from crime scene to forensic evidence in *Criminal and Environmental* Soil *Forensics* (eds Ritz, K. Dawson, L. & Miller, D.) 105–127 (Springer Science + Business Media B.V., 2009).

[8] Forster N, Grave P (2012). Non-destructive PXRF analysis of museum-crated obsidian from the Near East. Journal of Archaelogical Science.

[9] Nampanya S, Suon S, Rast L, Windsor PA (2012). Improvements in smallholder farmer knowledge of cattle production, health and biosecurity in southern Cambodia between 2008 and 2010. Transboundary and Emerging Diseases.

[10] Stenberg B, Viscarra Rossel RA, Mouzan AM, Wetterlind J (2010). Visible and near infrared spectroscopy in soil science. Advances in Agronomy.

[11] McLaren T (2012). Rapid, non-destructive total elemental analysis of Vertisol soils using portable X-ray fluorescence (PXRF). Soil Science Society of America Journal.

[12] Tighe M (2018). The potential for portable X-ray fluorescence determination of soil copper at ancient metallurgy sites, and considerations beyond measurements of total concentrations. Journal of Environmental Management.

[13] Yang, H., Kuang, B. & Mouzan, A. M. Quantitative analysis of soil nitrogen and carbon at a farm scale using visible and near infrared spectroscopy coupled with wavelength reduction. *European Journal of Soil Science* **63** (2012).

[14] Kuang B, Mouzan AM (2012). Influence of the number of samples on prediction error of visible and near infrared spectroscopy of selected properties at the farm scale. European Journal of Soil Science.

[15] Viscarra Rossel RA, Behrens T (2010). Using data mining to model and interpret soil diffuse reflectance spectra. Geoderma.

[16] Geladi P, Kowalski BR (1986). Partial least-squares regression: a tutorial. Analytica Chimica Acta.

[17] Aitkenhead MJ, Coull MC, Dawson LA (2014). Predicting sample source location from soil analysis using neural networks. Environmental Forensics.

[18] Dawson LA, Hillier S (2010). Measurement of soil characteristics for forensic applications. Surface and Interface Analysis.

[19] Aitkenhead MJ, Owen M, Chambers DM (2012). Use of artificial neural networks in measuring characteristics of shielded plutonium for arms control. Journal of Analytical Atomic Spectrometry.

[20] Fitzpatrick, R. W. Soil: Forensic Analysis in *Wiley Encyclopedia of Forensic Science* (eds Jamieson, A. & Moenssens, A.) (John Wiley & Sons, 2009).

[21] Fanti, F., Bell, P. R., Tighe, M., Milan, L. A. & Dinelli, E. Geochemical fingerprinting as a tool for repatriating poached dinosaur fossils in Mongolia: A case study for the Nemegt Locality, Gobi Desert. *Palaeogeography, Palaeoclimatology, Palaeoecology accepted* 30 October 2017, 10.1016/j.palaeo.2017.10.032 (2018).

[22] Forster N, Grave P, Vickery N, Kealhofer L (2011). Non-destructive analysis using PXRF: methodology and application to archaeological ceramics. X-Ray Spectrometry.

[23] Singer, M. J. & Munns, D. N. *Soils: an Introduction*. 6 edn, (Pearson Prentice Hall, 2006).

[24] Wilding, L. P., Smeck, N. E. & Hall, G. F. *Pedogenesis and Soil Taxonomy. I. Concepts and Interactions*. Vol. 11A (Elsevier, 1983).

[25] Rawlins BG (2006). Potential and pitfalls in establishing the provenance of earth-related samples in forensic investigations. Journal of Forensic Sciences.

[26] R Development Core Team. *R: A language and environment for statistical computing*, http://www.R-project.org (2012).

[27] Maher BA (2016). Palaeoclimatic records of the loess/palaeosol sequences of the Chinese Loess Plateau. Quaternary Science Reviews.

[28] Nie J (2015). Loess Plateau storage of Northeastern Tibetan Plateau-derived Yellow River sediment. Nature Communications.

[29] Bird A (2015). Quaternary dust source variation across the Chinese Loess Plateau. Palaeogeography, Palaeoclimatology, Palaeoecology.

[30] Nie J, Song Y, King JW, Egli R (2010). Consistent grain size distribution of pedogenic maghemite of surface soils and Miocene loessic soils on the Chinese Loess Plateau. Journal of Quaternary Science.

[31] Nie J, Peng W (2014). Automated SEM–EDS heavy mineral analysis reveals no provenance shift between glacial loess and interglacial paleosol on the Chinese Loess Plateau. Aeolian Research.

[32] Lewis PAW, Stevens JG (1991). Nonlinear modeling of time series using multivariate adaptive regression splines (MARS). Journal of the American Statistical Association.

[33] Hengl T (2015). Mapping soil properties of Africa at 250 m resolution: Random Forests significantly improve current predictions. PLOS One.

[34] Windsor PA (2011). Perspectives on Australian animal health aid projects in South-East Asia. Transboundary and Emerging Diseases.

[35] Guedes A (2011). Characterization of soils from the Algrave region (Portugal): A multidisciplinary approach for forensic applications. Science and Justice.

[36] Mevik B, Wehrens R (2007). The pls Package: Principal Component and Partial Least Squares Regression in R. Journal of Statistical Software.

[37] Friedman JH (1991). Multivariate adaptive regression splines. Annals of Statistics.

[38] Milborrow, S. *earth: Multivariate adaptive regression spine models. Derived from mda:mars by Trevor Hastie and Rob Tibshirani. R package version 3.2-3*., http://CRAN.R-project.org/package=earth (2012).

[39] Crawley, M. J. *The R Book*. 3rd edn, (John Wiley and Sons, 2013).

[40] Borchers, H. W. *Savitzky-Golay smoothing - an R implementation*, https://stat.ethz.ch/pipermail/r-help/2004-February/045568.html (2004).

[41] Overmars KP, de Koning GHJ, Veldkamp A (2003). Spatial autocorrelation in multi-scale land use models. Ecological Modelling.

[42] Ribeiro PJ, Diggle PJ (2001). geoR: A package for geostatistical analysis. R-News.

