# Supplementary material for: Georeferenced soil provenancing with digital signatures
Source: Sci Rep. 2018 Feb 16;8:3162. doi: 10.1038/s41598-018-21530-7 (PMC5816621; doi:10.1038/s41598-018-21530-7)
Supplement: Supplementary file 1 — Supplementary Material [file 41598_2018_21530_MOESM1_ESM.doc]

**Supplementary Material**

**Georeferenced soil provenancing with digital signatures**

Tighe, M.^1^, Forster, N.^1^, Guppy, C.^1^, Savage, D.^2^, Grave, P.^3^, Young I.M.^1^

1. School of Environmental and Rural Science, University of New England, Armidale, NSW 2351, Australia

2. 138 Toms Gully Road, Black Mountain, NSW 2365, Australia.

3. Archaeomaterials Science Hub/Archaeology, University of New England, Armidale, NSW 2351, Australia

**Corresponding Author,* [*mtighe2@une.edu.au*](mailto:safazler2@une.edu.au)

**
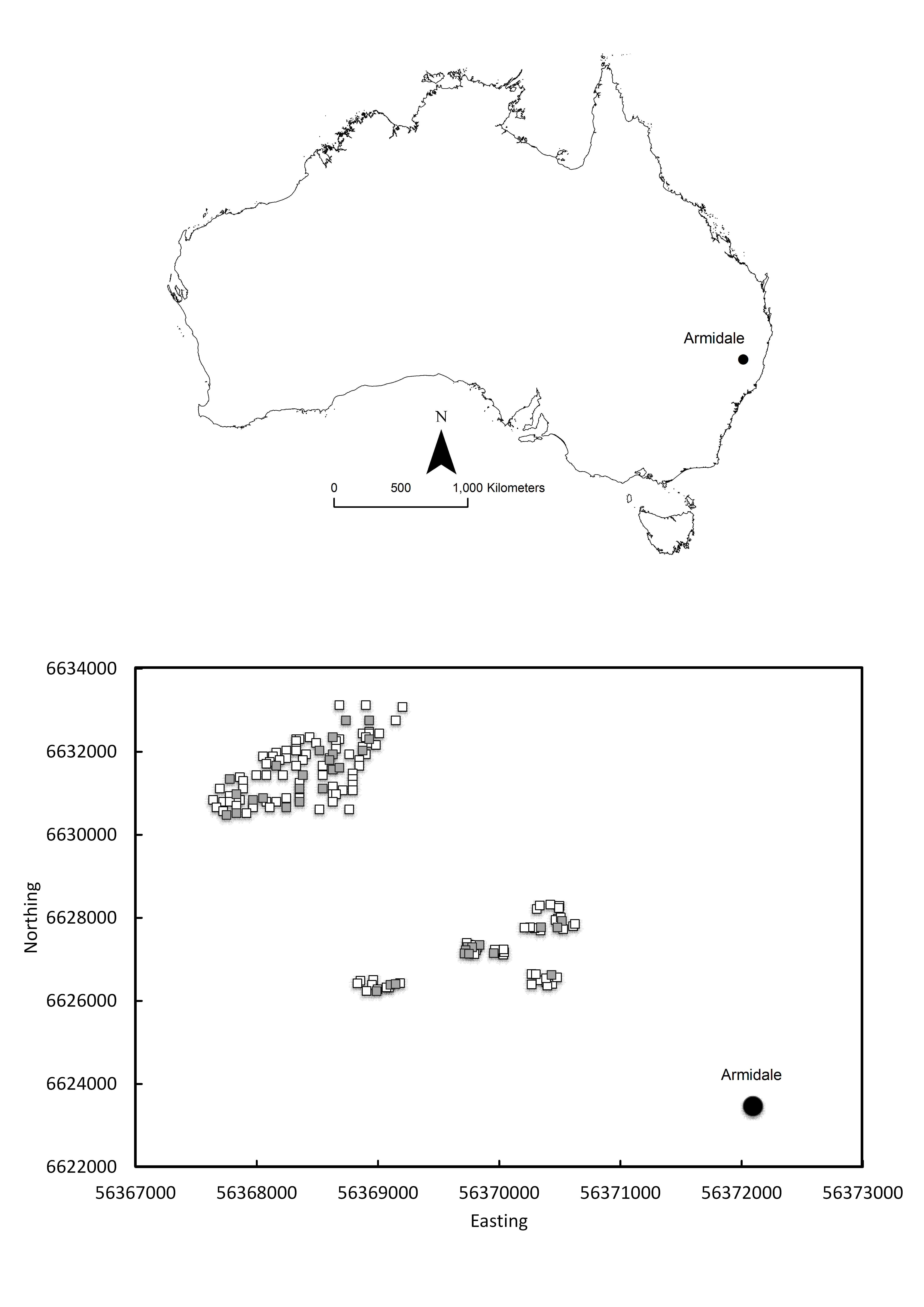
Supplementary Figure 1.** Study site location near Armidale, New South Wales (NSW), Australia (upper panel), and the location of individual samples (lower panel). Shaded sample points are the subset of samples for testing. Unshaded points are the subset of samples for method training. [Created with ArcGIS 10.2].

**Supplementary Figure 2.** Omnidirectional variograms (distance in metres) of the errors of prediction for Eastings (a) and Northings (b) using the Farm dataset, referencing the prediction approaches used to generate Figure 3. A small amount of spatial correlation is evident in predictions < approximately 400 m apart for the Eastings predictions, whereas no discernable pattern is evident in the Northings predictions. Variograms for the predictions summarized in Figure 4 at the Local dataset level show no spatial correlation and are not presented.
